# Pathophysiological Role of Variants of the Promoter Region of CITED2 Gene in Sporadic Tetralogy of Fallot Patients with Cellular Function Verification

**DOI:** 10.3390/biom12111644

**Published:** 2022-11-07

**Authors:** Zhuo Chen, Huan-Xin Chen, Hai-Tao Hou, Xiu-Yun Yin, Qin Yang, Guo-Wei He

**Affiliations:** 1 The Institute of Cardiovascular Diseases & Department Cardiovascular Surgery, TEDA International Cardiovascular Hospital, Tianjin University & Chinese Academy of Medical Sciences, Tianjin 300457, China; 2 School of Pharmacy, Drug Research & Development Center, Wannan Medical College, Wuhu 241002, China

**Keywords:** tetralogy of Fallot, CITED2, genetic, cardiac

## Abstract

Tetralogy of Fallot (TOF) is a common congenital heart malformation. Genetic variants in the CITED2 coding region are known to be significantly associated with cardiac malformation, but the role of variants in the CITED2 promoter region in the development of TOF remains unclear. In this study, we investigated CITED2 promoter variants in the DNA of 605 subjects, including 312 TOF patients and 293 unrelated healthy controls, by Sanger sequencing. We identified nine CITED2 gene promoter variants (including one novel heterozygous variant). Six were found only in patients with TOF and none in the control group. The transcriptional activity of the CITED2 gene promoter in mouse cardiomyocyte (HL-1) cells was significantly altered by the six variants (*p* < 0.05). The results of the electrophoretic mobility change assay and JASPAR database analysis showed that these variants generated or destroyed a series of possible transcription factor binding sites, resulting in changes in the CITED2 protein expression. We conclude that CITED2 promoter variants in TOF patients affect transcriptional activity and may be involved in the occurrence and progression of TOF. These findings may provide new insights into molecular pathogenesis and potential therapeutic insights in patients with TOF.

## 1. Introduction

Tetralogy of Fallot (TOF) is a common congenital heart malformation. It is the leading cause of cyanotic heart malformation in children [1], occurring in 3 out of every 10,000 live births and accounting for 7–10% of all congenital heart malformations [2]. Ventricular septal defect, right ventricular outflow tract obstruction, aortic overriding, and right ventricular hypertrophy are the basic pathological features [1]. Without timely surgical treatment, only 10% of patients can live to be over 20 years old [1]. With the improvement of surgical techniques, the mortality rate has been greatly reduced, but a large number of late morbidities, especially pulmonary valve insufficiency and atrial arrhythmias, are still important problems [3,4]. The pathogenesis of TOF involves many factors, and the etiology is still unclear. From the perspective of genetics, although a number of genetic variants have been identified, the genotype-phenotype relationship still needs to be further investigated [5,6,7,8,9]. Different genes often affect each other, and other factors, such as chromosomal abnormalities and related genetic syndromes, also play a role in the pathogenesis of TOF [6]. These factors are mixed together to form a very complex network system.

CITED2 (OMIM: 602937), the full name of Cbp/P300 interacting transactivation with Glu/Asp rich carboxy-terminal domain 2, is a protein-coding gene. CITED2 is a non-DNA-bound transcription coactivator and plays a key role in the development of embryonic and extra-embryonic tissues, such as heart morphogenesis [10]. In animal experiments, the embryonic death of CITED2-/- mice was accompanied by a variety of cardiac malformations [11], and HIF1 transcriptional activity was increased in CITED2 hearts and in CITED2 fibroblasts cultured under hypoxia conditions [12], suggesting that the loss of function of CITED2 may be one of the causes of cardiac septal malformations or defects. CITED2 variants were found in children with congenital heart disease (CHD), which was the first evidence to prove that CITED2 is the pathogenic gene for human congenital heart malformations. Subsequently, the important role of the regulation of CITED2 expression in different CHDs and its interaction with other nuclear proteins in early cardiac development and morphogenesis has been extensively studied [13,14,15,16].

However, those studies have focused on the coding region of CITED2, whereas the promoter region of CITED2, which is also critical for coordinating transcription within cells, has not been reported. DNA sequence variants in gene promoter regions may be associated with changes in gene expression levels, which may lead to diseases [17]. Based on the aforementioned, as well as on our study of the role of CITED2 in CHDs [18], we hypothesize that the change in CITED2 gene expression may be related to the promoter sequence variants that may play a role in the pathogenesis of TOF. In this study, we sequenced the CITED2 gene promoter from patients with sporadic TOF in comparison with healthy controls, and the identified variants were further tested in cellular function experiments.

## 2. Methods

### 2.1. Participants

**Figure 1:**
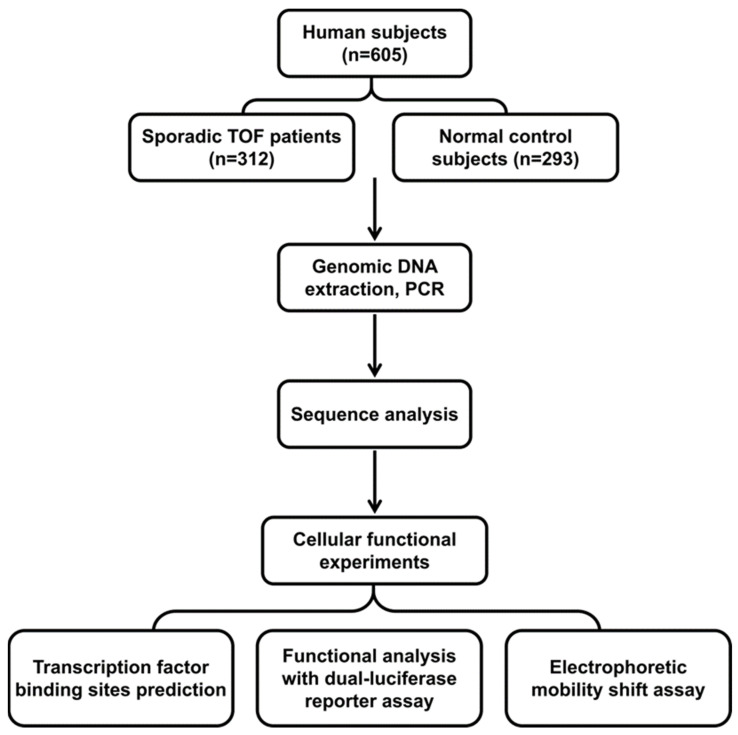
Study flow chart and experimental procedures. A total of 605 subjects participated in the study, including 312 tetralogy of Fallot (TOF) patients. Sequence analysis, cell function experiments, electrophoretic mobility shift analysis, and bioinformatics analysis were performed.

A total of 625 subjects were recruited in the study, including 312 isolated TOF patients and 293 healthy controls who had the same ethnicity and similar age. All patients were diagnosed with TOF based on clinical manifestations and echocardiography, and CT scans, in addition, when appropriate. The diagnosis was confirmed in all patients during corrective cardiac surgery at TEDA International Cardiovascular Hospital of Tianjin University, China. The controls were selected from routine health check-up or congenital heart disease screening programs. All control subjects were confirmed to have no family history of heart disease and other genetic disorders. In addition, neither physical examination nor echocardiography in the control subjects had heart defects or other major diseases (Figure 1). The study followed the principles of the Declaration of Helsinki and was approved by the Institutional Review Board of TEDA International Cardiovascular Hospital (Clinical Research Ethics Review Number: 2021-0715-4). Written informed consent was obtained from parents or guardians of all subjects.

### 2.2. Genomic DNA Extraction and Sequence Analysis

As we have reported [19,20], genomic DNA was extracted from the peripheral blood of each subject using a blood genomic DNA extraction kit. Primers were designed according to the CITED2 reference sequence (NCBI: NG_016169.1). The CITED2 promoter sequence (1418 bp, from −1197 bp to +220 bp from the transcription start point) and its flanking regions were obtained by PCR, and the product was sequenced directly. The primers required for PCR amplification and Sanger sequencing are shown in Table 1. The sequencing results were compared with the reference sequence by the software Chromas and DNAMAN to identify the variants.

### 2.3. Cell Level Validation

#### 2.3.1. Plasmid Construction

In the previous experiment [18], we amplified the wild-type fragment of the CITED2 gene promoter containing the restriction sites at the ends of KpnI and BglII. Through restriction endonuclease digestion, the wild-type subcloned into the KpnI/BglII site upstream of the firefly luciferase reporter gene plasmid (pGL3-Basic) to construct an expression vector and carry out Sanger sequence analysis (containing KpnI/BglII site The primers of the dots are shown in Table 1) to obtain the reporter plasmid pGL3 Basic-CITED2 promoter (WT) with the firefly luciferase reporter gene. In this experiment, the variants identified in the study were individually introduced into WT plasmids by using a site-directed gene mutagenesis kit according to the primer sequences in Table 1. DH5a cells were transfected and coated on LB plate. Positive clones were screened by Sanger sequencing, and plasmids were extracted by plasmid extraction kit.

#### 2.3.2. Cell Culture and Transfection

Mouse cardiomyocyte (HL-1) cells were thawed with DMEM supplemented with 10% fetal bovine serum, 1% 100 U/mL penicillin and streptomycin at 37 °C and 5% carbon dioxide. The passaged cells were cultured in six-well plates, and 24 h later, transfected with lipo2000. Transfection of the reporter plasmid with a reporter plasmid expressing Renilla luciferase (pRL-SV40) was used as an internal control to standardize the transfection efficiency. An empty pGL3 base vector was used as a negative control.

#### 2.3.3. Dual-Luciferase Reporter Gene Assay

**Figure 2:**
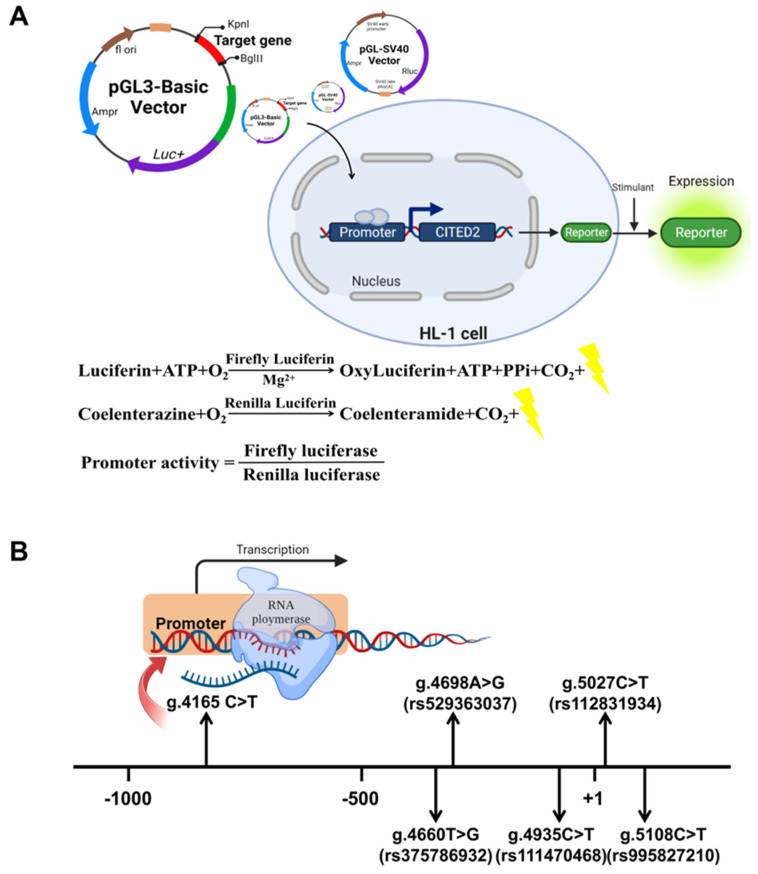
Relative transcriptional activity of the CITED2 gene promoter. (**A**) Procedure of cell function experiments. To confirm the effect of variants on promoter activity, wild-type and CITED2 gene promoter fragments with variants were prepared, gene expression vectors were constructed, and mouse cardiomyocyte (HL-1) cells were transfected. The transcriptional activity of wild-type and CITED2 gene promoters with variants was detected by a dual-luciferase reporter assay. (**B**) Regulatory variants of the CITED2 gene. The transcription start point is located at position 5001 of the first exon (+1), and these genetic variants are named according to the genomic sequence of the human CITED2 gene (Genbank Accession No. NG_016169.1), describing the location of the CITED2 gene promoter variants.

Cells were harvested and lysed 36–48 h after transfection. As shown in Figure 2A, luciferase activity was measured using a dual-luciferase reporter gene assay system (Thermo Scientific Fluoroskan FL, Waltham, MA, USA). The transcriptional activity of the CITED2 gene promoter was assessed by measuring the relative values of firefly luciferase activity and Renilla luciferase activity. The wild-type CITED2 gene promoter activity was set as 100%. The relative activity of the CITED2 gene promoter with variants was calculated. Experiments were independently repeated three times.

#### 2.3.4. Transcription Factor Binding Site (TFBs) Prediction

The JASPAR database (https://jaspar.genereg.net/), (accessed on 3 August 2022), with open access TF binding maps was used to further investigate whether the variants in the CITED2 promoter region would disrupt or generate TFBs [6,7]. The relative profile score threshold was set to 85%.

#### 2.3.5. Electrophoretic Mobility Shift Assay (EMSA)

Electrophoretic mobility shift assay (EMSA) was performed using a chemiluminescence EMSA kit. Nuclear proteins were extracted from HL-1 cells using a nuclear protein extraction kit. Double-stranded oligonucleotides (30 bp) were biotinylated as probes. After 20 min of incubation at room temperature, electrophoresis was performed on a 4% polyacrylamide gel at 90 V for 1 h, and the membrane was transferred at 330 mA. The nylon membrane was crosslinked with UV and the signal was detected by chemiluminescence.

### 2.4. Statistical Analysis

Statistical analysis was performed using IBM SPSS 28.0 software. For continuous variables, results are expressed as mean ± standard error (SEM). Quantitative data were compared using a standard Student’s *t*-test, and the statistical significance of the experimental results was calculated. The frequency of DNA sequence variants in patients with TOF and healthy controls was compared using Pearson’s chi-square test. Statistical analysis was performed using IBM SPSS 28.0 software for window trial (SPSS, Chicag, IL, USA).

## 3. Results

### 3.1. Genetic Variants Identified in the CITED2 Gene Promoter

**Figure 3:**
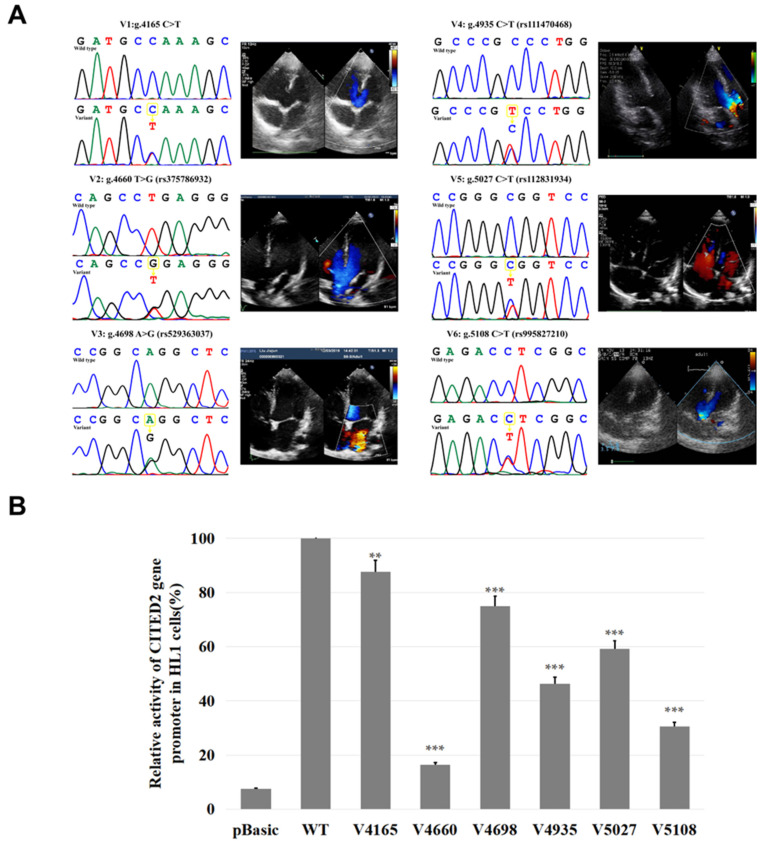
Sanger sequencing maps and color doppler echocardiography of variants. (**A**) The variants (V1-V6) of the CITED2 gene promoter found only in patients with TOF are shown [g.4165C>T, g.4660T>G(rs375786932), g.4698A>G(rs529363037), g.4935C>T(rs111470468), g.5108C >T (rs995827210), g.5027C>T (rs112831934)]. The top panel of V1–V6 shows the wild type, and the bottom panel shows the variant type. Next to each sequence map is the corresponding color doppler echocardiogram of the TOF patient that shows the major findings of those patients including a large malalignment ventricular septal defect and aortic overriding. (**B**) Relative transcriptional activity of the CITED2 gene promoter in HL-1 cells. The transcriptional activity of the wild-type CITED2 gene promoter was set as 100%, and the relative activity of the variants were calculated. Results are presented as the mean ± SD of three independent experiments, each in triplicate. Basic: empty vector; WT: wild type; V1: pGL3-4165T, V2: pGL3-4660G, V3: pGL3-4698G, V4: pGL3-4935C>T, V5: pGL3-4698G, V6: pGL3-5027C>T. **, *p* < 0.01; ***, *p* < 0.001.

Nine variants were identified in 605 subjects. Table 2 and Figure 2B summarize the distribution and location of the genetic variants. Of these nine variants, six variants were only found in TOF patients. Figure 3A shows the DNA sequencing chromatograms of these six variants. Among those variants, one (g.4165C>T) is a novel variant. The other five are reported SNPs in either gnomAD or 1000Genomes (https://www.ncbi.nlm.nih.gov/snp/), (accessed on 3 August 2022), as SNPs. However, the allele frequency of three [g.4698A>G(rs529363037), g.5108C>T(rs995827210), and g.5027C>T(rs112831934)] of these five variants is lower than 0.1%. The allele frequency of the other two variants [g.4660 T>G(rs375786932) and g.4935C>T(rs111470468)] is lower than 1%. However, in these NCBI databases, the allele frequency of the variant g.4935C>T(rs111470468) in East Asians is also lower than 0.1% (Table 2).

In contrast, the rest of three variants [g.4285T>G(rs12333191), g.4357G>A (rs76757432), and g.5122C>A(rs570422697)] were also found in the normal controls of this study (Table 2). Therefore, they were excluded from further cellular function studies [21].

### 3.2. Functional Analysis of Variants with Dual-luciferase Reporter Genes

The luciferase reporter gene expression vector was constructed with wild-type CITED2 gene promoter and the promoter with a variant, respectively. The expression vector of the wild-type CITED2 gene promoter was named pGL3-WT. The variants expression vectors included pGL3-4165T, pGL3-4660G, pGL3-4698G, pGL3-4935T, pGL3-5027T, and pGL3-5108T. The empty vector pGL3 was used as a negative control. Transfected HL-1 cells were collected and assayed for dual luciferase activity. The transcriptional activity of the wild-type CITED2 gene promoter was set as 100%. The relative transcriptional activities of the CITED2 gene promoter with a variant and the wild-type CITED2 gene promoter were calculated. As shown in Figure 3B, in HL-1 cells, all variants luciferase expressions were significantly lower than those of wild type (*p* < 0.05).

To determine whether genetic variants affect the putative binding sites of TFs, the CITED2 gene promoter was analyzed using the JASPAR database (http://jaspar.genereg.net/) (accessed on 3 August 2022) [19]. The results indicated that the reported five variants [g.4660T>G(rs375786932), g.4698A>G(rs529363037), g.4935C>T(rs111470468), g.5108C>T(rs995827210), and g.5027C>T(rs112831934)] may disrupt or generate potential TFBs. Table 3 summarizes the analytical data. As to the novel variant (g.4165C>T), because of its novelty, the JASPAR database is not able to predict the TFBs.

**Figure 4:**
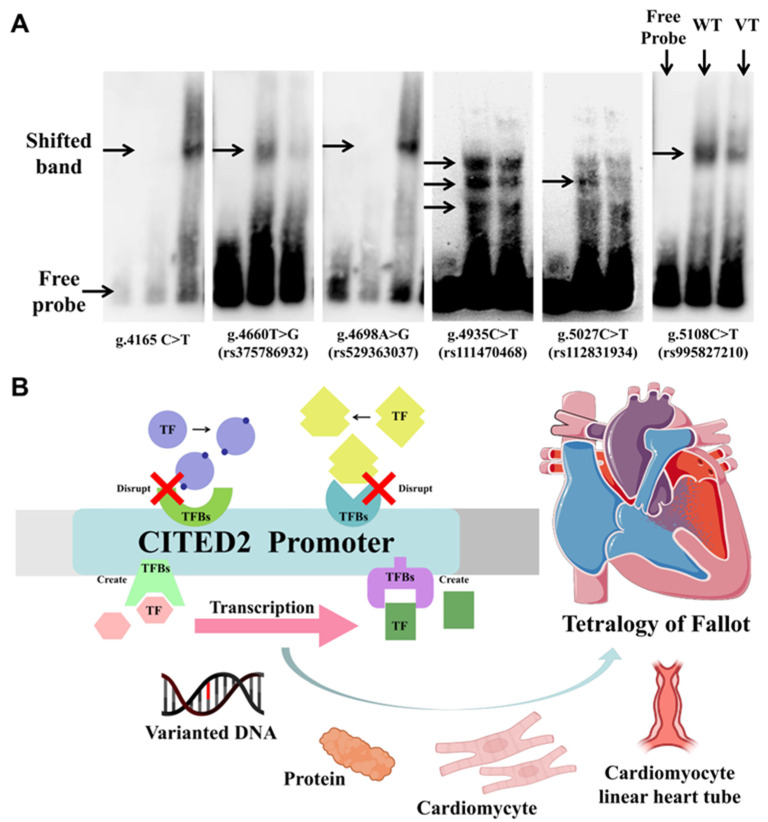
Electrophoretic mobility shift analysis (EMSA) experiments and schema on the effects of variants in the CITED2 promoter region on possible pathways in cardiac development. (**A**) Experimental results of electrophoretic mobility shift analysis of CITED2 gene promoter variants. Free probes are marked at the bottom; arrows indicate affected binding of unknown transcription factors (TFs); WT, wild type; VT, variant. (**B**) Effect of variants in the CITED2 promoter region on some TFs and the possible consequence of cardiac development.

In addition, EMSA experiments were used to qualitatively analyze whether genetic variants affect TFBs. The double-stranded biotinylated oligonucleotides used for EMSA are listed in Table 1. As shown in Figure 4A, all of these variants significantly altered the transcription factor binding.

## 4. Discussion

The present study, for the first time, identified the variants in the promoter region of the CITED2 gene in TOF patients in comparison with controls.

The human CITED2 gene is localized on chromosome 6q23.3 and is composed of two exons and two introns. Variants in the coding region of the CITED2 gene have been reported in a variety of human CHDs, including VSD, ASD, TOF, etc. [13,14,22]. However, there are no reports on the role of variants in the promoter region of the CITED gene in TOF. The CITED2 gene has an unusually large (3 kb) methylation modification site (CpG island) covering the promoter and transcription portion of the gene. The 5′ flanking region of this gene is active as a promoter in transient transfection assays and contains multiple STAT binding sites, consistent with its response to different cytokines [23]. In this study, the sequences of the CITED2 promoter region and its flanking regions were detected in 312 TOF patients. A novel sequence variant (g.4165C>T) and five SNPs (g.4660T>G, g.4698A>G, g.5108C>T, g.4935C>T, g.5027C>T) were identified in TOF patients. Importantly, these variants significantly affected the transcriptional activity of the CITED2 gene promoter by altering the TF binding. Interestingly, the variant g.4698A>G (rs529363037) identified in TOF patients in this study was also identified in isolated VSD patients in our previous study [18]. The fact that the variant g.4698A>G (rs529363037) is found in these two types of CHDs (TOF and isolated VSD) reveals the importance of this variant in the pathogenesis of CHDs. This point is further enhanced by the experiments performed on various types of cells, such as Hek-293, in previous studies and HL-1 in the present study. Those experiments have shown similar results, that the variant g.4698A>G (rs529363037) affected the TFBs in these different types of cells.

Normal heart development requires adequate gene doses of cardiac TFs. This has been demonstrated in both mouse and human studies, including NKX2-5, TBX5, GATA4, and TBX1. Those losses of function disrupt cardiac morphogenesis [13]. Figure 4B briefly depicts the possible mechanism of the pathological process of TOF with the involvement of the create/disrupt TFBs owing to the effect of the variants in the promoter region of CITED2.

In the results predicted by the JASPAR database in Table 3, CITED2 acts as a bridge connecting the neural crest transcription factor TFAP2 and the P300/CBP transcriptional co-activation complex, and the interaction of the three is crucial for the normal development of the cardiac neural crest [11]. E2F family members (E2F1 and E2F4) are involved in the regulation of CITED2 expression in neurons after stroke-related injury [24]. SP1, ELK1, ETV4, and STAT3 have also been confirmed to be involved in different pathways and cooperate with CITED2 or inhibit the occurrence of different diseases [25,26,27,28].

Genetic abnormalities involved in genes for heart development are increasingly being found to be associated with CHD in humans. Similar to other studies, monogenic genetic variants were associated with only a small proportion of CHD. Studies in larger populations with favorable phenotypes are needed to improve genotype-phenotype associations and to determine whether CITED2 variants are more common in TOF and other CHDs. Ultimately, further research is needed to elucidate the role of CITED2 in cardiac development to increase our understanding of the genetic basis of CHD, provide more personalized genetic counseling, and develop new preventive therapies.

## 5. Conclusions

In this study, we identified several genetic variants in the CITED2 gene promoter region in TOF patients. These genetic variants may alter the functional expression level of CITED2 by affecting TFBs to promote the development of TOF. These data provide a basis for further study of the molecular mechanism of CITED2 changes caused by genetic variants.

## Figures and Tables

**Table 1:** Primers used in this study.

Primer’s Name	Sequences 5′-3′	Location	Position
PCR and sequencing primers			
CITED2-F1	5′-AAAGGAAGAGTCCCAGCCGT-3′	3804	−1197
CITED2-F2	5′-TTTCTGCTCCGAAGACCGAG-3′	5221	+200
Primers containing restriction sites			
CITED2-KpnI ^a,b^	5′-(KpnI)-**GG**GGTACCAAAGGAAGAGTCCCAGCCGT-3′	3804	−1197
CCCAGCCGT-3′			
CITED3-BglII ^a,b^	5′-(BglII)-**CA**AGATCTTTTCTGCTCCGAAGACCGAG-3′	5221	+200
AGACCGAG-3′			
The double-stranded biotinylated oligonucleotides for the EMSA			
g.4165 C>T g.4660 T>G	5′-CTCAGCTCGGATGC(C/T)AAAGCTACCAAGAGCT-3′ 5′-TGGGGTAGATCCAGCC(T/G)GAGGGGGGCGGTGA-3′		
g.4698 A>G	5′-TCGTGGCTATCCCGGC(A/G)GGCTCTACCTTCGGGC-3′		
g.4935 C>T g.5027 C>T	5′-TTGGCGCCGCCCG(C/T)CCTGGAGGCTCTCG-3′ 5′-GGCAGCTGCCGGG(C/T)GGTCCTGCCGAGCT-3′		
g.5108 C>T	5′-CGGCCGCTGCGAGAC(C/T)TCGGCGCCGACATCG-3′		

Note: PCR primers are designed based on the genomic DNA sequence of the CITED2 gene (NG_016169.1). The transcription start site is at the position of 5001 (+1). Abbreviations: F, upstream primer; ^a^ Limit position underscore. ^b^ Protective bases are shown in bold.

**Table 2:** Genetic variants of CITED2 gene promoter in TOF patients and controls.

Variants	TOF ^a^	Controls ^a^	Position ^b^	Genotypes	Allele Frequency ^c^		Study
Frequency in Control = 0 (Further Validation)					Global	East Asian	
g.4165C>T	1	0	−836 bp	CT	-	-	-
g.4660 T>G(rs375786932)	1	0	−341 bp	TG	C = 0.000167	C = 0.0067	gnomAD-Genomes
g.4698 A>G(rs529363037)	1	0	−303 bp	AG	C = 0.000072	C = 0.0029	gnomAD-Genomes
g.4935C>T(rs111470468)	1	0	−66 bp	CT	A = 0.015720	A = 0.0003	gnomAD-Genomes
g.5108 C>T(rs995827210)	1	0	+107 bp	CT	A = 0.000100	A = 0.0000	gnomAD-Genomes
g.5027C>T (rs112831934)	1	0	+26 bp	CT	A = 0.0002	A = 0.0000	1000Genomes
**Frequency in Control ≠ 0 (No Further Validation)**							
g.4285T>G(rs12333191)	27	33	−716 bp	TG	C = 0.231281	C = 0.0467	gnomAD-Genomes
g.4357G>A(rs76757432)	1	16	−644 bp	GA	T = 0.094422	T = 0.0006	gnomAD-Genomes
g.5122C>A(rs570422697)	1	1	+121 bp	CA	T = 0.000029	T = 0.0013	gnomAD-Genomes

Abbreviations: −, not applicable; TOF, tetralogy of Fallot. ^a^ Allele frequency in groups. ^b^ Variants are located upstream (-) to the transcription start site at the position of 5001 (+1) of CITED2 gene (NG_016169.1). ^c^ Allele frequencies from East Asians in the NCBI dbSNP database.

**Table 3:** Effects of the promoter region variants of the CITED2 gene on TFBS predicted by JASPAR database.

Variants	Binding Sites for Transcription Factors		Promoter Activity
	Create	Disrupt	
g.4165 C>T	-	-	↓
g.4660 T>G (rs375786932)	MAZ, ZNF148, ZNF462, ZNF740	ZNF736	↓
g.4698 A>G (rs529363037)	STAT3, ZNF780A, ZNF548, ZNF273, ZNF462	THAP1, TFAP2E, ZNF783	↓
g.4935C>T (rs111470468)	ZNF354C, MAZ, EHF, ETV4	KLF14, KLF2, KLF5, KLF6, NR2C2, SP1, SP2, SP4, TFAP2B, ZNF704	↓
g.5027C>T (rs112831934)	ZNF354C, ERF::NHLH1, ZNF273, NR2C2, YBX1	RFX5, THAP1, ZNF704	↓
g.5108C >T (rs995827210)	E2F1, SOX18	PAX5, ZBTB21, ZNF571	↓

Abbreviations: -, not applicable; TFBS, transcription factor binding sites.

## Data Availability

The individual SNP numbers are given in Table 2. The genetic variants described in this manuscript are available at https://www.ncbi.nlm.nih.gov/snp/ (accessed on 3 August 2022). Data supporting the findings of this study are available upon reasonable request from the corresponding authors.
